# Diagnostic yield and limitations of whole-genome sequencing for hereditary cerebellar ataxia

**DOI:** 10.1093/braincomms/fcaf188

**Published:** 2025-05-17

**Authors:** Wai Yan Yau, Roisin Sullivan, Emer O’Connor, David Pellerin, Michael H Parkinson, Paola Giunti, Marie-Josée Dicaire, Matt C Danzi, Stephan Züchner, Bernard Brais, Nicholas W Wood, Henry Houlden, Jana Vandrovcova

**Affiliations:** Department of Neuromuscular Diseases, UCL Queen Square Institute of Neurology, University College London, London WC1N 3BG, United Kingdom; Perron Institute for Neurological and Translational Science, the University of Western Australia, Nedlands, Western Australia 6009, Australia; Department of Neuromuscular Diseases, UCL Queen Square Institute of Neurology, University College London, London WC1N 3BG, United Kingdom; Department of Neuromuscular Diseases, UCL Queen Square Institute of Neurology, University College London, London WC1N 3BG, United Kingdom; Department of Neuromuscular Diseases, UCL Queen Square Institute of Neurology, University College London, London WC1N 3BG, United Kingdom; Ataxia Centre, UCL Queen Square Institute of Neurology, University College London, London WC1N 3BG, UK; National Hospital for Neurology and Neurosurgery, University College London Hospitals NHS Foundation Trust, London WC1N 3BG, UK; Ataxia Centre, UCL Queen Square Institute of Neurology, University College London, London WC1N 3BG, UK; National Hospital for Neurology and Neurosurgery, University College London Hospitals NHS Foundation Trust, London WC1N 3BG, UK; Department of Neurology and Neurosurgery, Montreal Neurological Hospital and Institute, McGill University, Montreal, QC, Canada, H3A 2B4; Dr John T Macdonald Foundation Department of Human Genetics and John P. Hussman Institute for Human Genomics, University of Miami Miller School of Medicine, Miami, FL 33136, USA; Dr John T Macdonald Foundation Department of Human Genetics and John P. Hussman Institute for Human Genomics, University of Miami Miller School of Medicine, Miami, FL 33136, USA; Department of Neurology and Neurosurgery, Montreal Neurological Hospital and Institute, McGill University, Montreal, QC, Canada, H3A 2B4; Department of Human Genetics, McGill University, Montreal, QC, Canada, H3A 2B4; National Hospital for Neurology and Neurosurgery, University College London Hospitals NHS Foundation Trust, London WC1N 3BG, UK; Department of Clinical and Movement Neurosciences, UCL Queen Square Institute of Neurology, London WC1N 3BG, United Kingdom; Department of Neuromuscular Diseases, UCL Queen Square Institute of Neurology, University College London, London WC1N 3BG, United Kingdom; National Hospital for Neurology and Neurosurgery, University College London Hospitals NHS Foundation Trust, London WC1N 3BG, UK; Department of Neuromuscular Diseases, UCL Queen Square Institute of Neurology, University College London, London WC1N 3BG, United Kingdom

**Keywords:** ataxia, neurogenetics, whole-genome sequencing, movement disorders

## Abstract

Less than half of the individuals with hereditary cerebellar ataxia receives a genetic diagnosis. Repeat expansions account for disproportionate number of hereditary cerebellar ataxia and have genetically heterogeneous causes. These genetic loci include *ATXN1*, *ATXN2*, *ATXN3*, *CACNA1A*, *ATXN7*, *ATXN8OS*, *ATXN10*, *PPP2R2B, TBP*, *ATN1*, *FMR1, BEAN1, NOP56, GLS, THAP11, GAA-FGF14, ZFHX3, FXN* and *RFC1.* This study aims to assess the yield of short-read whole genome sequencing in the molecular diagnosis of hereditary cerebellar ataxia. We recruited 380 patients (351 probands) from a national ataxia centre in United Kingdom. They underwent short-read whole genome sequencing as a part of the 100 000 Genomes Project. Bioinformatic pipeline of whole genome sequencing include variant prioritization in selected virtual gene panels, customized analysis with a focus on repeat expansions, structural variants and recently reported hereditary cerebellar ataxia genes. All potential genetic variants were reviewed in a multidisciplinary team, and further confirmation tests were performed as appropriate. Whole genome sequencing identified causative variants in 115 (33%) out of 351 probands. We established 46 distinct presumptive molecular diagnoses with the most frequent being *SPG7* (*n* = 22)*, RFC1* (*n* = 20) and *CACNA1A* (*n* = 10). However, it failed to detect any probands with novel ataxia gene *GAA-FGF14*, which was subsequently identified on polymerase chain reaction screening in 10 unsolved probands. In conclusion, whole genome sequencing is a useful diagnostic test in hereditary cerebellar ataxia patients and can be used to detect repeat expansions, structural and mitochondrial variants. However, identification of complex structural variants and sizing of large repeat expansions remains a challenge and require alternative molecular testing techniques.

## Teaser

Yau *et al*. report that short-read whole genome sequencing in genetically pre-screened cohort of hereditary cerebellar ataxia achieved a diagnostic yield of 33%. These molecular diagnoses include repeat expansions, structural and mitochondrial variants. However, short-read whole genome sequencing failed to detect novel ataxia gene *GAA-FGF14*.

## Graphical Abstract

**Graphical Abstract:**
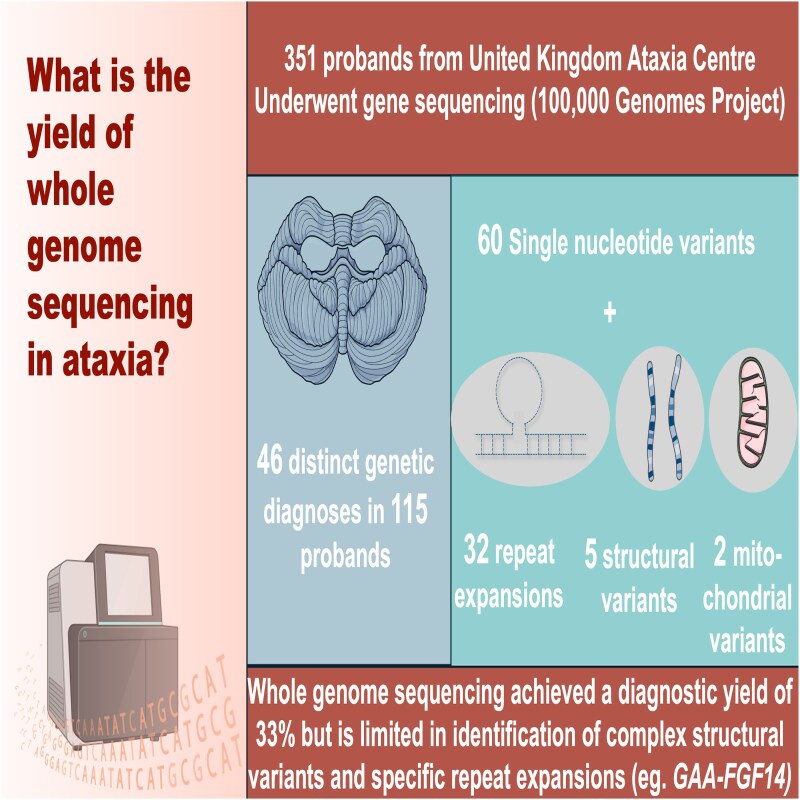

## Introduction

Hereditary cerebellar ataxias (HCA) are progressive neurodegenerative diseases that share the clinical features of ataxia due to cerebellar degeneration. Estimated prevalence of this group of diseases reaches 11 in 100 000 people.^1^ There are over a hundred genetic loci responsible for HCA.^2,3^ Whilst patients with specific manifestations such as oculomotor apraxia or vestibular areflexia infer underlying genetic aetiologies,^4-6^ the utility of clinical-genetic classification in HCA is limited by high level of phenotype–genotype overlap. Traditional genetic diagnostic techniques such as Sanger sequencing, repeat-primed polymerase chain reaction (RP-PCR) and Southern blotting are increasingly superseded by short-read massive parallel sequencing. However, targeted genetic panel and whole exome sequencing in HCA have a diagnostic ceiling at 50%^7^; limited data are available on the diagnostic yield of whole genome sequencing (WGS).^8^ WGS has the potential to improve diagnostic yields further, by improved ability to detect variants in non-coding regions and the mitochondrial genome, expansion variants and more robust detection of structural variants. Identifying a molecular diagnosis may lead to gene-specific therapy and facilitate accurate genetic counselling and family planning. The 100 000 Genomes Project (100 kGP) was set up to introduce and embed genomic testing into the mainstream National Health Service (NHS), discover new disease genes and make genetic diagnosis available for more patients in the United Kingdom (UK).^9^ Here, we report the diagnostic yields for 380 HCA patients recruited into the main programme between 2015 and 2020 at a national ataxia referral service.

## Materials and methods

### Participants

We enrolled patients with suspected HCA and their family members recruited to the 100 kGP between 2015 and 2020 from the National Hospital of Neurology and Neurosurgery (NHNN) UK. Prior to 100 kGP recruitment, all patients had received diagnostic tests available in the NHS based on clinicians’ discretion without reaching a molecular diagnosis. These included PCR-based repeat expansion analyses (*ATXN1, ATXN2, ATXN3, ATXN7, CACNA1A, TBP, ATN1, FXN, HTT or C9Orf72)*, single gene analysis, mitochondrial testing, gene panel and/or whole exome sequencing. We retrieved the clinical information against a standardized list of clinical features through patients’ medical records/ patient assessment (Supplementary Table 1). Patients were excluded if subsequent clinical diagnoses were deemed not caused by a genetic aetiology. We classified the patients into seven clinical subgroups modified from the study by Coutelier *et al*.:^10^ (i) pure ataxia phenotype, (ii) spastic ataxia, (iii) metabolic presentation with mitochondrial features (sensorineural hearing loss, ptosis, ophthalmoplegia, optic atrophy, retinal dystrophy, leukodystrophy), (iv) sensory ataxia, (v) complex early-onset ataxia with additional neurological features not otherwise classified (age at onset <30 years), (vi) complex late-onset clinical picture with additional neurological features not otherwise classified (age at onset ≥30 years), (vii) Episodic ataxia antecedent to progressive ataxia. Autosomal dominant inheritance was considered plausible when there is vertical transmission whilst recessive inheritance was supported by family history of consanguinity or at least 2 affected siblings. Joint ethics committee of UCL Queen Square Institute of Neurology, and NHNN approved this study (UCLH: 04/N034).

### Whole genome sequencing and research pipeline

The details of WGS and standard bioinformatic pipelines have been previously outlined.^9^ In brief, DNA extracted from peripheral blood was sequenced to a national specification (Illumina TruSeq, HiSeq 2500 and HiSeq X). Reads were aligned to the Genome Reference Consortium human genome build 37 and/or 38 using Isaac Genome alignment software and later Dragen pipelines. Family-based variant calling of single-nucleotide variants and indel was performed using Platypus variant caller. Short nucleotide variants were classified by the 100 kGP into tiered groups using virtual gene panels. Gene panels were selected from curated ‘PanelApp’ based on patients’ HPO terms.^11^ Variants outside of curated panels were prioritised using Exomiser for further manual curation. We also retrieved variants in recently identified causative ataxia genes i.e.*SPTAN1*, *NPTX1*, *PNPT1, SAD9L*.^12-15^ Copy number variants (CNV) and structural variants (SV) generated by 100 kGP using Canvas and MANTA; only CNVs and SVs in known HCA genes were analysed. Variants were annotated and prioritized using allele frequencies in the 100 kGP and gnomAD datasets. Short tandem repeat expansion genotyping was performed using ExpansionHunter v3.0.0.^16-21^ for 27 loci (*HTT, AR, ATN1, ATXN1, ATXN2, ATXN3, ATXN7, CACNA1A, ATXN8OS, ATXN10, TBP, C9orf72, FXN, FMR1, DMPK, PPP2R2B, CNBP, CSTB, JPH3, BEAN1, NOP56, NOTCH2NLC, GLS, THAP11, ZFHX3, RFC1* and *FGF14*). For *RFC1,* AAGGG_(n)_ and ACAGG_(n)_ were considered as pathogenic motifs. Mitochondrial variants and their heteroplasmy were called using Mutect2. Known pathogenic variants based on confirmed status in Mitomap were selected for further follow-up.

Within the setting of a multidisciplinary team, we reviewed these variants to determine their clinical significance based on the ACMG classification, inheritance pattern, clinical fit between patients’ phenotypes and the reported phenotypes for the gene. Further segregation studies and Sanger sequencing validations were carried out. When WGS detected a repeat expansion, a confirmatory Polymerase Chain Reaction and/or Southern Blot Analysis was performed. Due to a strong suspicion that WGS analysis had missed *GAA-FGF14*, we performed validated PCR tests in 14 unsolved probands.^22,23^ These probands were in the top 5th percentile of the unsolved probands (*n* = 236) for repeat expansion size estimated by ExpansionHunter (range: 166–211 GAA repeats).

### Statistical methods

Comparisons between two groups were tested for statistical significance using a χ^2^ test for qualitative variables. Association between the overall diagnostic yield or yield of routine diagnostic pipeline versus customized re-analysis (outcome variables) and various clinical and genetic factors (dependent variables) were tested using logistic regression. Bonferroni correction was used for *P*-values. Analyses were performed using R version 3.6.1.

## Results

### Demographics and clinical characteristics

**Figure 1:**
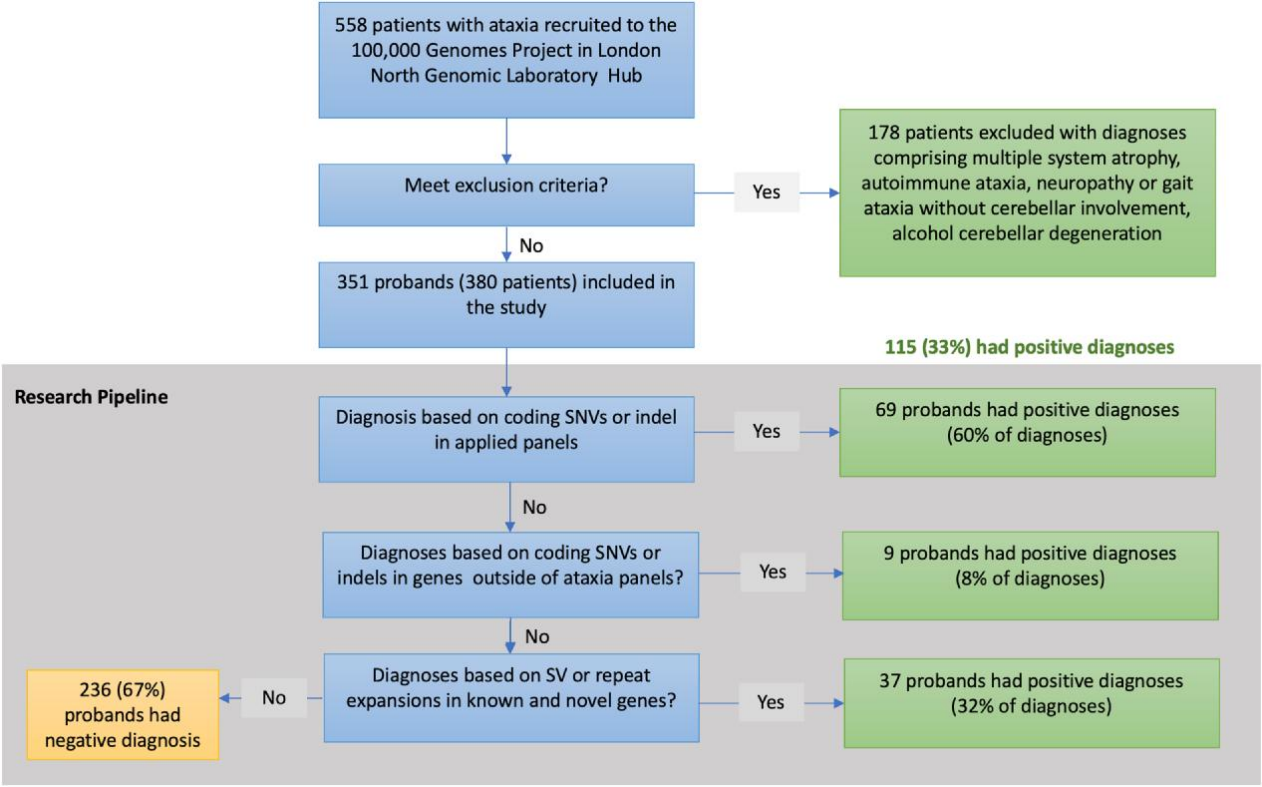
**Overview of the diagnostic and research pipeline and source of diagnoses.** Results from 380 probands with hereditary cerebellar ataxia showed that a total of 33% of the probands received a positive genetic diagnosis. SNV, single nucleotide variant; SV, structural variant.

This study focused on a cohort of patients seen at the NHNN Neurogenetics Unit and Ataxia Centre between January 2015 and October 2020, and who were recruited to the 100 kGP for WGS (Fig. 1). In total, our cohort included 380 individuals with a clinical diagnosis of hereditary ataxia from 351 families. Most patients were recruited to the 100 kGP and their genetic analyses conducted as singletons (72%), followed by duos (13%) and trios (11%). The most common ancestry of enrolled probands was White British (79%) and South Asians (9.2%). There was no gender difference (male = 190; female = 190). Positive family history of cerebellar ataxia was reported for 41% of all patients. Of these, 53% had probable autosomal dominant inheritance pattern whilst 42% were probable autosomal recessive.

### Clinical details of hereditary ataxia cohort

In this cohort of unsolved HCA, we analysed the clinical characteristics of 380 individuals with HCA enrolled in the study. A third of the cohort had age at ataxia onset before 30 years. The predefined clinical subgroups comprised 108 pure ataxia (28%), 63 spastic ataxia (17%), 31 sensory ataxia (8%), 56 ataxia with metabolic features (15%), 40 early complex ataxia (11%), 59 late complex ataxia (16%) and 23 episodic ataxia (6%). Whilst 330 affected individuals (87%) demonstrated cerebellar oculomotor signs, only a small proportion of them had external ophthalmoplegia and/or ptosis (4%) or oculomotor apraxia (1%). More than two-third of the cohort had additional neurological and non-neurological features associated with ataxia; co-morbid neurological features most often seen were spasticity (28%), cognitive impairment (22%), peripheral neuropathy (19%), urinary dysfunction (19%) and tremor (15%). Abnormalities were also common on neuroimaging. The most frequent features were cerebellar atrophy (60%), cortical atrophy (13%), small vessel disease (13%), brainstem atrophy (10%), leukoencephalopathy (5%) and thin corpus callosum (3%). The cohort underwent prior genetic testing: repeat expansion analysis (90%), mitochondrial testing (38%), single genetic tests (24%), gene-panel analysis (16%) and whole-exome sequencing (1%). Table 1 outlines the details of the subgrouping and clinical characteristics of the cohort.

**Table 1:** Ataxia clinical subgroups and their demographics, clinical characteristics, and genetic results

	Ataxia clinical subgroups							Entire ataxia cohort (*N* = 380)
	Pure (*N* = 108)	Spastic (*N* = 63)	Sensory (*N* = 31)	Metabolic (*N* = 56)	Early complex (*N* = 40)	Late complex (*N* = 59)	Episodic (*N* = 23)	
Demographics								
Sex								
Female	54 (50%)	34 (54%)	21 (68%)	26 (46%)	19 (48%)	29 (49%)	7 (30%)	190 (50%)
Male	54 (50%)	29 (46%)	10 (32%)	30 (54%)	21 (53%)	30 (51%)	16 (70%)	190 (50%)
Ancestry								
European	97 (90%)	55 (87%)	28 (90%)	40 (71%)	32 (80%)	49 (83%)	22 (96%)	323 (85%)
Asian	6 (6%)	6 (10%)	2 (6%)	7 (13%)	7 (18%)	8 (14%)	0 (0%)	36 (9%)
Black Caribbean	1 (1%)	1 (2%)	0 (0%)	2 (4%)	1 (3%)	2 (3%)	0 (0%)	7 (2%)
Middle Eastern	0 (0%)	0 (0%)	1 (3%)	5 (9%)	0 (0%)	0 (0%)	0 (0%)	6 (2%)
Others^a^	4 (4%)	1 (2%)	0 (0%)	2 (4%)	0 (0%)	0 (0%)	1 (4%)	8 (2%)
Age, median in years (range)	65 (20–92)	60 (25–90)	73 (30–90)	60 (24–83)	39 (6–75)	70 (37–89)	66 (23–84)	63 (6–92)
Positive family history^b^	46 (43%)	21 (33%)	8 (26%)	29 (52%)	19 (48%)	20 (34%)	13 (57%)	156 (41%)
Ataxia clinical characteristics								
Age at onset^c^								
<30 years	19 (18%)	17 (27%)	5 (17%)	29 (52%)	40(100%)	0 (0%)	10 (48%)	120 (32%)
≥30 years	84 (82%)	45 (73%)	25 (83%)	27 (48%)	0 (0%)	59 (100%)	11 (53%)	250 (66%)
Ophthalmic signs								
Cerebellar^d^	91 (84%)	57 (90%)	28 (90%)	48 (86%)	35 (88%)	52 (88%)	19 (83%)	330 (87%)
Ophthalmoplegia	0 (0%)	0 (0%)	0 (0%)	13 (23%)	0 (0%)	0 (0%)	0 (0%)	13 (3%)
OMA	0 (0%)	0 (0%)	0 (0%)	2 (4%)	2 (5%)	1 (2%)	0 (0%)	5 (1%)
Other movement disorders								
Tremor^e^	0 (0%)	6 (10%)	3 (10%)	9 (16%)	17 (43%)	20 (34%)	1 (4%)	56 (15%)
Parkinsonism	0 (0%)	1 (2%)	0 (0%)	1 (2%)	3 (8%)	13 (22%)	0 (0%)	18 (5%)
Myoclonus	0 (0%)	0 (0%)	0 (0%)	2 (4%)	6 (15%)	6 (10%)	0 (0%)	14 (4%)
Chorea	0 (0%)	0 (0%)	0 (0%)	2 (4%)	5 (13%)	4 (7%)	0 (0%)	11 (3%)
Dystonia	0 (0%)	3 (5%)	1 (3%)	3 (5%)	19 (48%)	17 (29%)	0 (0%)	43 (11%)
Non-movement disorder related symptoms								
Cognitive impairment	14 (13%)	14 (22%)	4 (13%)	20 (36%)	14 (35%)	18 (31%)	1 (4%)	85 (22%)
Pyramidal signs	0 (0%)	63 (100%)	3 (10%)	23 (41%)	14 (35%)	4 (7%)	0 (0%)	107 (28%)
Epilepsy	0 (0%)	0 (0%)	1 (3%)	6 (11%)	3 (8%)	12 (21%)	0 (0%)	22 (6%)
Peripheral neuropathy	0 (0%)	5 (8%)	31 (100%)	13 (23%)	9 (23%)	13 (22%)	1 (4%)	72 (19%)
Dysautonomia	0 (0%)	2 (3%)	5 (16%)	4 (7%)	0 (0%)	6 (10%)	0 (0%)	17 (4%)
Vestibular impairment	0 (0%)	1 (2%)	8 (26%)	0 (0%)	1 (3%)	2 (3%)	1 (4%)	13 (3%)
Hypogonadotrophic hypogonadism	0 (0%)	0 (0%)	0 (0%)	1 (2%)	2 (5%)	1 (2%)	0 (0%)	4 (1%)
Optic atrophy/retinal dystrophy	0 (0%)	0 (0%)	0 (0%)	24 (43%)	0 (0%)	0 (0%)	0 (0%)	24 (6%)
Urinary dysfunction	24 (22%)	17 (27%)	5 (16%)	9 (16%)	5 (13%)	13 (22%)	0 (0%)	73 (19%)
Brain MRI abnormality	80 (74%)	48 (76%)	24 (77%)	44 (79%)	28 (70%)	45 (76%)	10 (43%)	279 (73%)
Previous genetic tests								
Repeat expansion analysis	98 (91%)	58 (92%)	29 (94%)	49 (88%)	38 (95%)	57 (97%)	13 (57%)	342 (90%)
Mitochondrial testing	30 (28%)	20 (32%)	10 (32%)	34 (61%)	22 (55%)	25 (42%)	5 (22%)	146 (38%)
Single gene analysis^f^	20 (19%)	18 (29%)	6 (19%)	13 (23%)	16 (40%)	14 (24%)	6 (26%)	93 (24%)
Gene-panel analysis	8 (7%)	20 (32%)	6 (19%)	9 (16%)	8 (20%)	4 (7%)	5 (22%)	60 (16%)
WES	0 (0%)	2 (3%)	1 (3%)	1 (2%)	1 (3%)	0 (0%)	0 (0%)	5 (1%)
Diagnostic rate (proband only)								
Proband number	*N* = 98	*N* = 60	*N* = 31	*N* = 51	*N* = 36	*N* = 55	*N* = 20	351 (92%)
Genetically solved (total)	10 (10%)	25 (42%)	20 (65%)	24 (47%)	13 (36%)	16 (29%)	7 (35%)	115 (33%)

Data are n/N (%); percentage may not add up to 100% due to rounding.

OMA, oculomotor apraxia.

^a^Ashkenazi Jews, Moroccans, Slavic, mixed White and South Asian.

^b^Reported to have first-degree or second-degree relatives with ataxia.

^c^Missing *n* = 9.

^d^Gaze evoked nystagmus, interrupted pursuit, hypermetric saccades, hypometric saccades, downbeating nystagmus.

^e^Upper limb/head/palatal tremor.

^f^Includes Prion, Ataxia with oculomotor apraxia and episodic ataxia gene testing.

### Diagnostic yield

**Figure 2:**
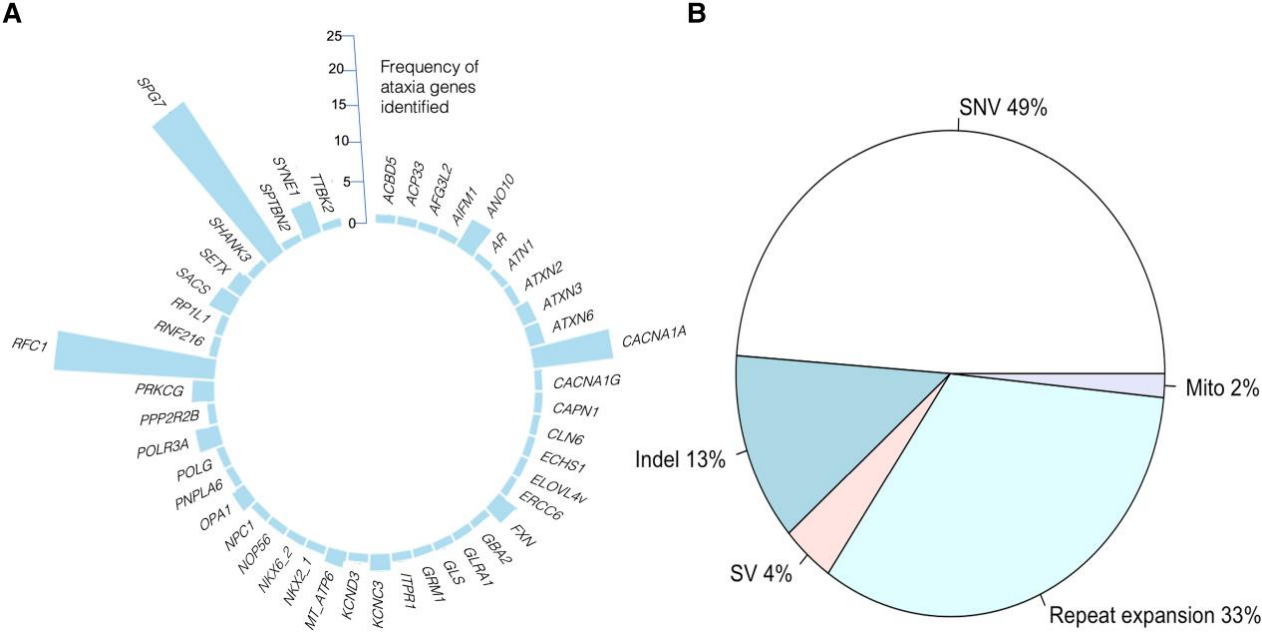
**Molecular diagnostic results for the hereditary cerebellar ataxia cohort.** (**A**) Diagnostic gene frequency identified in ataxia cohort. (**B**) Gene variant type in percentage. Indel, insertion-deletion; Mito, mitochondrial variant; SNV, single nucleotide variant; SV structural variant.

The diagnostic yield of WGS in this cohort of 351 unsolved HCA probands was 33%. We established 46 distinct presumptive molecular diagnoses in 115 probands (Fig. 2A and Supplementary Table 2). The genetic variant type comprised 60 single nucleotide variants (49%), 32 repeat expansions (33%), 16 indels (13%), 5 SV (4%) and 2 mitochondrial variants (2%) (Fig. 2B and Supplementary Table 3). The expanded repeats were found in these ataxia genes: *RFC1* (homozygous), *ATN1*, *ATXN2*, *ATXN3*, *ATXN6*, *FXN* (homozygous), *NOP56, PPP2R2B.* No proband carried the newly identified repeat expansion in *GAA-FGF14*, *THAP11* or *ZFHX3*. Structural variants were identified in *ANO10*, *SPG7* and *GLS*; both mitochondrial variants were found in *MT-ATP6*. However, genetic diagnoses of two families (76 and 121) were missed at the time of initial analysis in 2021 because (i) hereditary ataxia panel had not been updated to include *NKX2_1*; (ii) the splice variant *POLR3A* c.1909 + 22G > A was filtered out by the bioinformatic pipeline as a synonymous variant.

**Figure 3:**
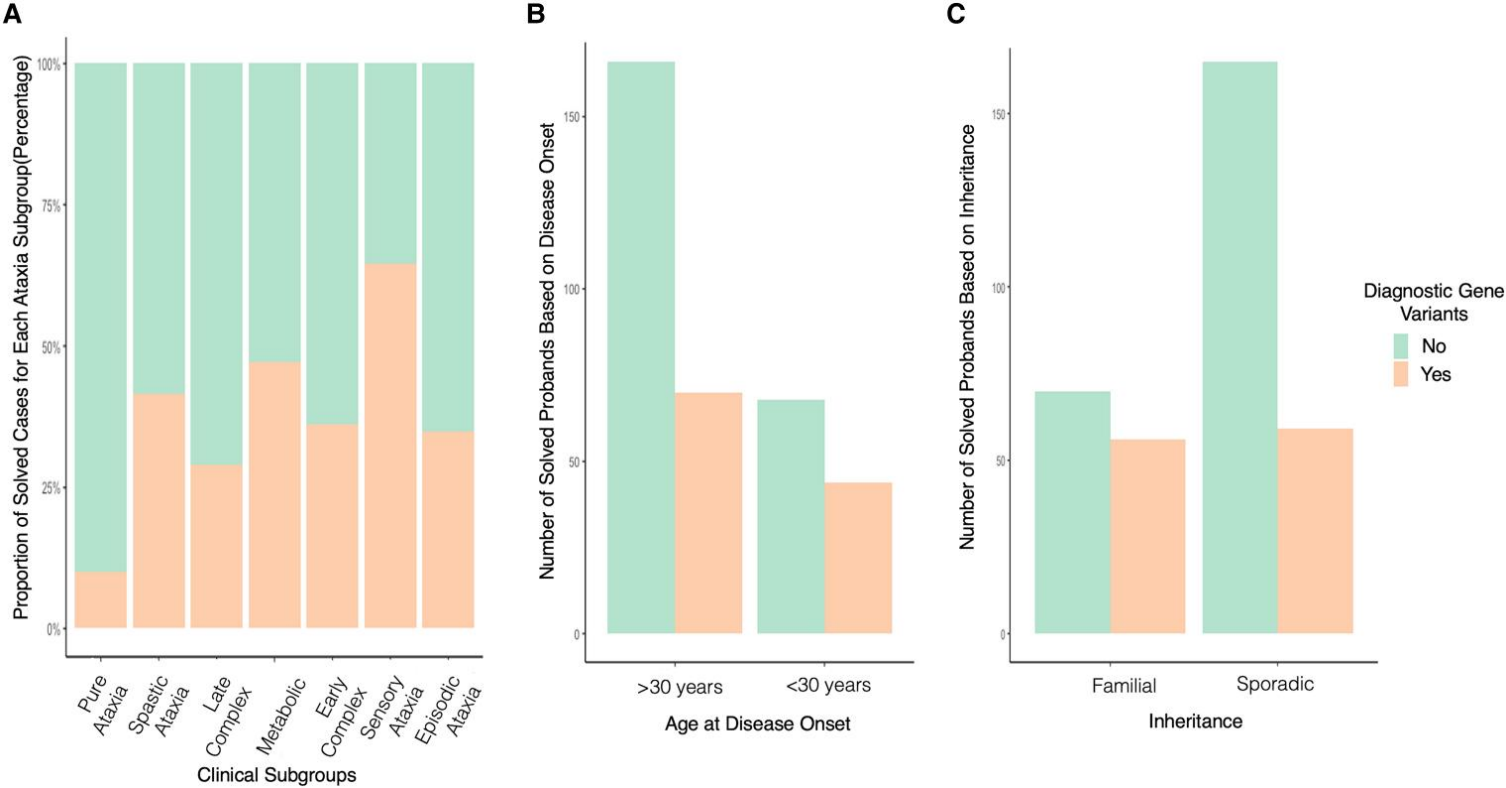
**Clinical factors that influence hereditary cerebellar ataxia gene identification.** (**A**) Proportion of probands with diagnostic gene variants for different clinical subgroups; (**B**) Age at disease onset; (**C**) Family history.

To identify factors that influence the diagnostic yield of WGS, we examine the association between ataxia clinical subgroup, age at disease onset and family history (Fig. 3). The diagnostic yield for ataxia subgroups in descending order were sensory ataxia (65%), ataxia with metabolic features (47%), spastic ataxia (42%), early complex ataxia (36%), episodic ataxia (35%), late complex ataxia (29%) and pure ataxia (10%). Probands receiving a positive genetic diagnosis were twice as likely to have a family history than those without a family history (95% CI: 1.4–3.6; *P* = 0.0005; Supplementary Table 4). A positive genetic diagnosis may be associated with earlier age of disease onset although this was not statistically significant (*P* = 0.07). In the parsimonious model, lack of family history (*P* = 0.014) and clinical subgroup of pure ataxia (*P* < 0.0001) remained statistically significant negative predictors for achieving a genetic diagnosis and clinical subgroup of sensory ataxia (*P* = 0.018) was a positive predictor.

### Genotype–phenotype association

The predefisned clinical group was generally concordant with the molecular diagnoses (Supplementary table 5). The most common causative genes identified in each clinical subgroups were: *CACNA1A* (*n* = 2) in pure ataxia; *SPG7* (*n* = 13) in spastic ataxia; *RFC1* (*n* = 17) in sensory ataxia; *SPG7* (*n* = 6) in ataxia with metabolic features; *ATXN3, RFC*, *SPG7* (*n* = 2 each) in late complex ataxia; and *CACNA1A* (*n* = 6) in episodic ataxia. Early complex ataxia subgroup did not have the same diagnostic variant in the same gene in more than two probands. WGS expanded phenotypic spectrum in *ERCC6* (chorea-ataxia) and *ECHS1* (attenuated form with independent ambulation) as illustrated in Supplementary Text 1 and Supplementary Fig. 1. The diagnosis of seven probands with genes not included in the hereditary ataxia panel were made by selecting additional gene panels on Genomics England PanelApp based on detailed clinical phenotyping with HPO terms (Supplementary Table 6).

### GAA-FGF14 testing

WGS did not identify any probands with *GAA-FGF14* repeat expansion above pathogenic threshold of 250 repeats using ExpansionHunter. However, we performed PCR tests and were able to detect 10 probands out of 14 who carried a heterozygous GAA repeat expansion in the pathogenic range (Supplementary Table 7). The average repeat size of allele one and two were 69 (range: 8–206) and 352 (range: 260–427). Nine out of 14 probands screened had episodic ataxia. Nine had GAA repeat expansion size above 250 whilst one had GAA repeat expansion size of 213.

## Discussion

A comprehensive analysis of short-read WGS data improves genetic diagnostic yield of hereditary cerebellar ataxia (HCA). Important advantages of WGS are its ability to simultaneously interrogate multiple genomic regions of interest, better coverage of non-coding regions and mitochondrial genomes, detection of repeat expansions and SV. In our study, repeat expansions account for nearly a third of the genetic diagnosis in our study and bioinformatic tool such as ExpansionHunter has been proven to be useful in identifying neurological repeat expansion disorders and assists to differentiate the continually expanding list of normal and pathogenic variations of the repeat motifs.^21,22,24,25^

In comparison to previous studies in which large numbers of HCA patients were investigated with whole exome sequencing (WES), the diagnostic yields ranged from 20 to 33.5%.^7,10,26-28^ We achieved a diagnostic yield of 33% and our cohort includes predominantly unsolved probands. The majority of our cohort underwent PCR tests prior to WGS to exclude common trinucleotide repeat expansion ataxia such as SCA1, SCA2, SCA3, SCA6, SCA7, SCA12 and SCA17. Our study also has the largest HCA-specific cohort that underwent WGS to the best of our knowledge. The high diagnostic rate of sensory ataxia subgroup was driven by the detection of *RFC1* homozygous repeat expansion. Our study also reinforces the clinical and genetic heterogeneity of HCA, with 46 distinct molecular diagnoses identified and outlined phenotypic expansion of *ERCC6* and *ECHS1*. This is consistent with the study outcome by Cunha *et al.* demonstrating the extreme phenotypic heterogeneity in 756 patients with non-expansion spinocerebellar ataxia.^29^

However, there are important caveats associated with WGS use in neurogenetic disorders.^30^ Firstly, repeat expansions with size above the usual read length of short-read WGS is not identified in WGS. Failure to detect *GAA-FGF14* in our study using WGS is an example of this shortfall. Independent research groups have demonstrated bioinformatic tools such as ExpansionHunter and STRling have the potential as screening tools for *GAA* repeat expansions in *FGF* when coupled with deep phenotyping.^31,32^ Furthermore, ExpansionHunter can also be applied to whole exome sequencing data or gene panel.^33^ This may produce similar diagnostic yield in a more cost-effective manner depending on resource availability and local expertise. Secondly, short-read WGS also struggles to detect variants in pericentromeric, sub-telomeric regions, copy-neutral SV, segmental duplications and GC-rich simple repetitive elements. Long-read WGS or optical genome mapping have improved diagnostic accuracy for repeat expansion and structural variants.^31,34,35^ Incorporation of these novel technologies with short-read WGS will further improve HCA diagnostic yield. Thirdly, assessing pathogenicity of variants of unknown significance (VUS) in noncoding regions remains a major challenge due to incomplete gene ontological databases for these genomic variations. Multi-omics approach with integration of RNA-seq and proteomics data is a potentially useful strategy. The multidisciplinary team approach to unsolved cases with clinicians with specialist genetic knowledge and diagnostic genomic scientist will also help resolve some of these VUS and ensure appropriate genes and panels are included for testing. Lastly, the ability of gene panels in WGS to detect recently identified ataxia genes depends on contemporaneous updates of the panels based on literatures. This was demonstrated by the missed diagnoses of *NKX2-1* and *POLR3A* in two families in our study from initial analysis.

### Limitations

Individuals recruited to this study were selected from a national referral ataxia service and may be predisposed to selection bias. This may also impact on the external validity of our data. Furthermore, our patient cohort did not receive uniform genetic tests prior to enrolment to this study. They underwent genetic investigations selected by the referring clinicians: over 90% had repeat expansion testing but only 16% had genetic panel. We did not have information on whether the study participants had previous research-based whole exome sequencing. Therefore, our study can only make indirect comparison between WGS, WES, and ataxia panel. Many of the molecular diagnoses, especially single nucleotide variants, identified in this study could have been achieved with ataxia panel or whole exome sequencing. In addition, a recent study by Rafehi *et al*. reported that ExpansionHunter achieved a sensitivity of 100% with a false positive rate of only 16% for screening of *GAA-FGF14* repeat expansion when an empirical threshold of >90 (GAA)_n_ was applied.^31^ We would have missed patients carrying this repeat expansion or other repeats with expansion size above 250 base pairs. Lastly, we only considered AAGGG_(n)_ and ACAGG_(n)_ as pathogenic motifs for homozygous repeat expansions in *RFC1.* Motifs AGGGC_(n)_, AAGGC_(n)_, AGAGG_(n)_ and large AAAGG (≥600 repeats) were later identified to be pathogenic,^25^ which were not known at the time of our study's genomic data analysis. Thereby, we would have misclassified patients carrying these repeat motifs as benign.

In summary, our study shows that WGS is a useful diagnostic test for patients with HCA. However, a large proportion of patients with HCA do not receive a genetic diagnosis after WGS. Ongoing future research focus on gene discovery using novel sequencing and multi-omics technologies will help narrow this diagnostic gap.

## Supplementary Material

fcaf188_Supplementary_Data

## Data Availability

The supporting findings of this study are available from the corresponding author upon reasonable request. All codes were saved in the Genomics England research environment. Unfortunately, access to the code is restricted in the 100 000 Genomes Project and our request for public access was declined because the code used in this project included participant ID and results involving phenotypic data counts below five. Please see specific restrictions outlined: http://re-docs.genomicsengland.co.uk/airlock/ and https://re-docs.genomicsengland.co.uk/airlock_rules.
