# Supplementary material for: Diagnostic yield and limitations of whole-genome sequencing for hereditary cerebellar ataxia
Source: Brain Commun. 2025 May 17;7(3):fcaf188. doi: 10.1093/braincomms/fcaf188 (PMC12142459; doi:10.1093/braincomms/fcaf188)
Supplement: fcaf188_Supplementary_Data [file fcaf188_supplementary_data.pdf]

Supplementary Text 1. Examples of genotype-phenotype in the genes *ERCC6* and *ECHS1.*

We identified compound heterozygous variants in *ERCC6* (ENST00000355832: c.2167C>T; p. Gln723*; ENST00000355832:c.1820A>T:p.Lys607Met) in patient A (patient 119 in supplementary table 2): a 64 year-old woman manifested with progressive cerebellar ataxia, chorea and spasticity from age 41 years. Her exposed skin showed mild lentigines and both feet had ichthyosis. Her MRI brain showed posterolateral putamina mineralisation and global atrophy including brainstem and cerebellum (supplementary figure 1C-F). Her sister was similarly affected (family tree in supplementary figure 1A). p.Gln723* in *ERCC6* is a stop-gained variant that was reported to be pathogenic. The second variant p.Lys607Met causes a missense change with minor allele frequency of 0.000063 in gnomAD.(Gudmundsson *et al.*, 2022) In-silico analyses predict this variant to be probably damaging in polyphen and deleterious in SIFT. These variants in patient A were in trans and were identified in her affected sister.

In patient B (patient 117 in supplementary table 2), we identified compound heterozygous variants in *ECHS1* (ENST00000368547.4:c.299T>C; ENST00000368547.4: c.518C>T). He is a 62-year-old man with autosomal recessive cerebellar ataxia, sensorineural hearing loss, optic atrophy and pendular nystagmus (supplementary figure 1B). His MRI head demonstrated bilateral T2-hyperintensive changes in globus pallidi and mild superior cerebellar volume loss (supplementary figure 1 G-J). His sister is similarly affected. Their age at disease onset were in early childhood. C.299T>C in *ECHS1* is a missense variant that was reported to be pathogenic. The second variant c.518C>T is also a missense variant that is absent in gnomAD and computationally predicted to have a deleterious effect. These variants are in trans.

ERCC6 encodes an effector protein excision repair 6 chromatin remodelling factor and is a part of nucleotide excision repair (NER) pathway targeting helix-distorting lesions in DNA; recessive pathogenic variants in *ERCC6* cause Cockayne syndrome B.(Kraemer *et al.*, 2007) Biallelic variants of another genes in the NER pathway *ERCC4* lead to the same milder phenotype of our patient.(Doi *et al.*, 2018b) Cordts *et al.* recently coined adult-onset neurodegeneration in nucleotide excision repair disorders and presented 13 patients with biallelic variants in genes involved in nucleotide excision pathways (*ERCC4, ERCC2* and *XPA).(Cordts et al., 2022)* All individuals have similar clinical phenotype, neuroimaging findings as in our patient with inconspicuous dermatological manifestations. This is further supported by another case series of four Japanese patients with biallelic variants in *ERCC4.(Doi et al., 2018a)* *ECHS1* encodes the mitochondrial enoyl-CoA hydratase which catalyses the fourth degradation step of the branched-chain amino acid valine but also takes part in the beta-oxidation of short-chain fatty acids. Biallelic pathogenic variants in *ECHS1* were reported to cause early-onset Leigh-like syndrome and dystonia-ataxia syndrome.(Peters *et al.*, 2014; Ronchi *et al.*, 2020) All other reported cases with *ECHS1* variants were severely disabled from childhood with the oldest reported surviving patient at 31 years old and was confined to wheelchair from 9 years due to tetraplegia.(Haack *et al.*, 2015) Our patient is the oldest surviving individual without severe intellectual impairment and remains ambulant.

References:

Cordts I, Onder D, Traschutz A, Kobeleva X, Karin I, Minnerop M*, et al.* Adult-Onset Neurodegeneration in Nucleotide Excision Repair Disorders (NERD(ND)): Time to Move Beyond the Skin. Mov Disord 2022; 37(8): 1707-18.

Doi H, Koyano S, Miyatake S, Nakajima S, Nakazawa Y, Kunii M*, et al.* Cerebellar ataxia-dominant phenotype in patients with ERCC4 mutations. J Hum Genet 2018a; 63(4): 417-23.

Doi H, Koyano S, Miyatake S, Nakajima S, Nakazawa Y, Kunii M*, et al.* Cerebellar ataxia-dominant phenotype in patients with ERCC4 mutations. J Hum Genet 2018b.

Gudmundsson S, Singer-Berk M, Watts NA, Phu W, Goodrich JK, Solomonson M*, et al.* Variant interpretation using population databases: Lessons from gnomAD. Hum Mutat 2022; 43(8): 1012-30.

Haack TB, Jackson CB, Murayama K, Kremer LS, Schaller A, Kotzaeridou U*, et al.* Deficiency of ECHS1 causes mitochondrial encephalopathy with cardiac involvement. Ann Clin Transl Neurol 2015; 2(5): 492-509.

Kraemer KH, Patronas NJ, Schiffmann R, Brooks BP, Tamura D, DiGiovanna JJ. Xeroderma pigmentosum, trichothiodystrophy and Cockayne syndrome: a complex genotype-phenotype relationship. Neuroscience 2007; 145(4): 1388-96.

Peters H, Buck N, Wanders R, Ruiter J, Waterham H, Koster J*, et al.* ECHS1 mutations in Leigh disease: a new inborn error of metabolism affecting valine metabolism. Brain 2014; 137(Pt 11): 2903-8.

Ronchi D, Monfrini E, Bonato S, Mancinelli V, Cinnante C, Salani S*, et al.* Dystonia-ataxia syndrome with permanent torsional nystagmus caused by ECHS1 deficiency. Ann Clin Transl Neurol 2020; 7(5): 839-45.

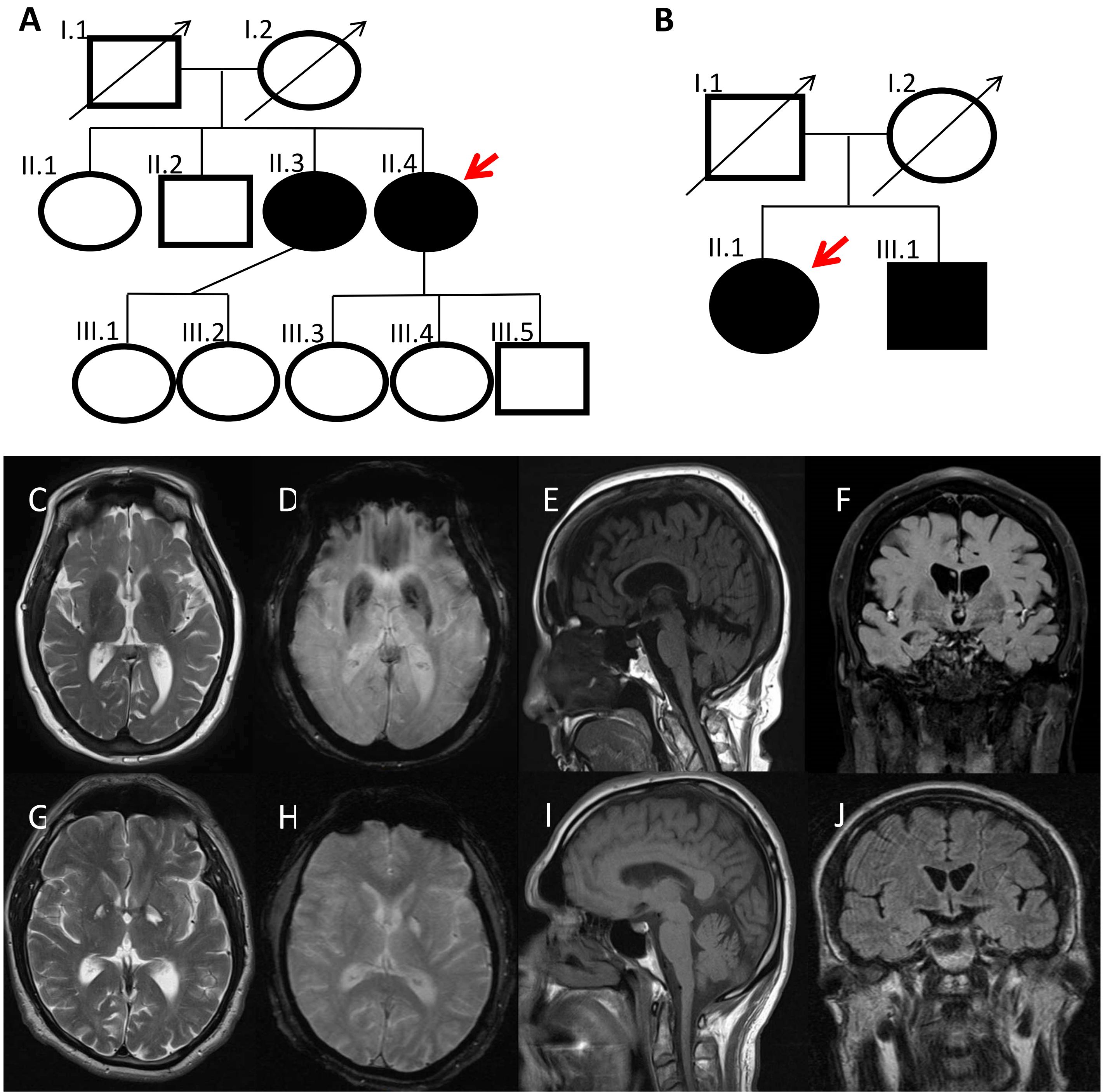

Supplementary Figure 1.
Family trees of patients with *ERCC6* and *ECHS1*: Probands with *ERCC6* (A) and *ECHS1* (B) putative variants were indicated by red arrows.
MRI brain sequences of proband with *ERCC6* variants: axial T2 (C), axial SWI (D), sagittal T1 (E) and coronal T1 (F). These panels showed prominent mineralization of the posterolateral aspects of the putamina on T2 and SWI sequence and generalized parenchymal volume loss without disproportionate regional atrophy.
MRI brain sequences of proband with *ECHS1* variants: axial T1 (G), axial SWI (H), sagittal T1 (I) and coronal T2 Flair (J). These panels showed focal T2 hyperintense changes involving bilateral globus pallidi and mild superior cerebellar volume loss.

Supplementary table 1. Clinical data proforma		
**Demographics**		**Patient ID**
**Sex**		
**Ancestry**		
	European	
	Asian	
	Black Caribbean	
	Middle Eastern	
	Others	
**Age at review**		
**Deceased**		
**Positive family history**		
	Autosomal dominant	
	Autosomal recessive	
	Mitochondrial	
	Sporadic	
**Ataxia clinical characteristics**		
**Age at onset**		
**Ophthalmic signs**		
	Cerebellar eye signs	
	Ophthalmoplegia/ ptosis	
	Oculomotor apraxia	
**Other movement disorder(s)**		
**Tremor**		
**Parkinsonism**		
**Myoclonus**		
**Chorea**		
**Dystonia**		
**Non-movement disorder related symptoms**		
**Developmental delay**		
**Cognitive impairment**		
**Pyramidal signs**		
**Epilepsy**		
**Peripheral Neuropathy**		
	Sensory predominant	
	Motor predominant	
	Mixed	
	Slow conduction velocity	
**Dysautonomia**		

**Vestibular impairment**	
**Hypogonadotrophic hypogonadism**	
**Optic atrophy/ retinal dystrophy**	
**Kyphoscoliosis**	
Urinary dysfunction	
Auditory neuropathy	
Cataract	
Migraine	
Cough	
Pes cavus	
Rem sleep disorder	
Other parasomnia	
**Brain MRI abnormality**	
	Cerebellar atrophy
	Brainstem atrophy
	Leukodystrophy
	Corpus callosum atrophy
	Mineralisation
	Iron deposition
	Cortical atrophy
	Small vessel disease
	Hot cross bun sign
**Previous genetic testing**	
	Repeat expansion analysis
	Mitochondrial testing
	Ataxia with oculomotor apraxia testing
	Episodic ataxia testing
	Prion testing
	Gene-panel analysis
	WES
**Sequencing methods (WGS)**	
	Solo
	Duo
	Trio
	Quartet
	Quintet

Supplementary Table 2 Probands with pathogenic/ likely pathogenic variants and “hot” variants of unknown significance identified through WGS

ID; gender; age	Ataxia Subgroup	Gene	cDNA variant	Zygosity	Predicted consequence	Estimated consequence as per ACMG	Allele frequency in Gnomad	Variants previously reported to be disease causing	CADD	Sift	Poly-phene	Z- score	GERP	Co-segregation evidence
**1a: F, 31**	Early complex	*NKX6-2*	ENST00000368592.8:c.121A>T	Hom	Nonsense	Likely pathogenic	0	yes	36				0.22	yes
**1b: M, 29**	Early complex	*NKX6-2*	ENST00000368592.8:c.121A>T	Hom	Nonsense	Likely pathogenic	0	yes	36				0.22	yes
**2a: M, 71**	Metabolic	*SPG7*	ENST00000645818.2:c.1529C>T	Compound Het	Splice acceptor	Pathogenic	0.003588	yes	25.4			1.2	3.45	yes
			ENST00000645818.2:c.1454_1462del		missense	Pathogenic	0.0004022	yes					3.45	
**2b: F, 79**	Metabolic	*SPG7*	ENST00000645818.2:c.1529C>T	Compound Het	Splice acceptor	Pathogenic	0.003588	yes	25.4			1.2	3.45	yes
			ENST00000645818.2:c.1454_1462del		missense	Pathogenic	0.0004022	yes					3.45	
**3: M, 40**	Early complex	*AR*	ENST00000396044:c.189_239dup51	Het	Repeat expansion	Pathogenic		yes						
**4a: F, 51**	Spastic ataxia	*FXN*	ENST00000484259.3:c.165+1357_165+1358ins(GAA)75 ; ENST00000484259.3:c.165+1357_165+1358ins(GAA)101	Hom	Repeat expansion	Pathogenic		yes						yes
**4b: F, 48**	Spastic ataxia	*FXN*	ENST00000484259.3:c.165+1357_165+1358ins(GAA)75 ; ENST00000484259.3:c.165+1357_165+1358ins(GAA)101	Hom	Repeat expansion	Pathogenic		yes						yes
**5: M, 42**	Metabolic	*RNF216*	ENST00000389902.8:c.2453-2A>G	Hom	Splice acceptor	Likely pathogenic	0	yes						
**6: F, 73**	Late complex	*RFC1*	*RFC1*	Hom	Repeat expansion	Pathogenic		yes						no
**7: M, 70**	Spastic ataxia	*SPG7*	ENST00000645818.2:c.739C>T	Hom	Nonsense	Likely pathogenic	0.00001314	yes	25.4					no
**8: M, 61**	Spastic ataxia	*SPG7*	ENST00000645818.2:c.1529C>T	Compound Het	Splice acceptor	Likely pathogenic	0.003588	yes	25.4			1.2	3.45	no
			ENST00000645818.2:c.806G>A		Nonsense	Pathogenic	0	yes						
**9: F, 70**	Sensory ataxia	*RFC1*	*RFC1*	Hom	Repeat expansion	Pathogenic		yes						no
**10: F, 54**	Pure	*ATXN6*	*ATXN6*	Het	Repeat expansion	Pathogenic		yes						no
**11: F, 55**	Spastic ataxia	*SYNE1*	ENST00000367255.10:c.18644G>A	Compound Het	Nonsense	Likely pathogenic	0	no						no
			ENST00000367255.10:c.1828G>T		Nonsense	Likely pathogenic	0	no						
**12: F, 54**	Pure	*PPP2R2B*	*PPP2R2B*	Het	Repeat expansion	Pathogenic		yes						no
**13: M, 62**	Episodic ataxia	*CACNA1A*	ENST00000360228.11:c.2633_2649dup	Het	Frameshift-->stop gained	Likely pathogenic	0	no						no

**14: M, 40**	Spastic ataxia	*SYNE1*	ENST00000367255.10:c.16111C>T	Compound Het	Nonsense	Likely pathogenic	0	yes						no
			ENST00000367255.10:c.24312+8C>G		Splice region		0.000006571	yes	0.585			1.78	-1.31	
**15: F, 76**	Sensory ataxia	*RFC1*	*RFC1*	Hom	Repeat expansion	Pathogenic		yes						no
**16: F, 78**	Sensory ataxia	*RFC1*	*RFC1*	Hom	Repeat expansion	Pathogenic		yes						no
**17: F, 27**	Spastic ataxia	*SETX*	ENST00000224140.6:c.6163_6167del	Hom	Frameshift	Likely pathogenic	0	no						no
**18: M, 55**	Early complex	*GBA2*	ENST00000378103.7:c.1687+1G>A	Hom	Splice donor	Likely pathogenic	0	no						no
**19: M, 74 (deceased)**	Late complex	*RFC1*	*RFC1*	Hom	Repeat expansion	Pathogenic		yes						no
**20: M, 62**	Metabolic	*SPG7*	ENST00000645818.2:c.1454_1462del	Compound Het	Splice acceptor	Likely pathogenic	0.0004022	yes	32			1.2	3.45	no
			ENST00000645818.2:c.1672A>T		Nonsense	Likely pathogenic	0.00009197	yes	48					
**21: M, 64**	Metabolic	*SACS*	ENST00000382292.9:c.9305T>A	Hom	Nonsense	Pathogenic	0.000006572	yes	42				2.32	no
**22: M, 57**	Spastic ataxia	*SPG7*	ENST00000645818.2:c.1529C>T	Hom	Missense	Pathogenic	0.003588	yes	25.4			1.2	3.45	no
**23: M, 76**	Pure	*ELOVL4*	ENST00000369816.5:c.698C>T	Het	Missense	Likely pathogenic	0	yes	26.7	0	0.972		2.48	yes
**24: M, 69**	Metabolic	*OPA1*	ENST00000361510.8:c.2779-1G>T	Het	Splice region	Pathogenic	0	no						no
**25: M, 55**	Pure	*KCNC3*	ENST00000477616.2:c.1259G>A	Het	Missense	Pathogenic	0	yes						no
**26: M, 56**	Spastic ataxia	*SPG7*	ENST00000645818.2:c.15_23dup	Hom	Structural variant-Dup	Likely pathogenic	0	no						no
**27: M, 24**	Early complex	*NPC1*	ENST00000269228.10:c.3493G>A	Hom	Missense	Likely pathogenic	0.00001314	yes	28.1				1.28	no
		CCDC114	ENST00000674294.1:c.398del	Hom	Frameshift, incidental	Likely pathogenic	0	no						
**28: F, 79**	Late complex	*ATN1*	*ATN1*	Het	Repeat expansion	Pathogenic		yes						no
**29: F, 78**	Sensory ataxia	*RFC1*	*RFC1*	Hom	Repeat expansion	Pathogenic		yes						no
**30: M, 56**	Spastic ataxia	*SPG7*	ENST00000645818.2:c.1529C>T	Compound Het	Splice acceptor	Pathogenic	0.003588	yes	25.4			1.2	3.45	no
		SPG7	ENST00000645818.2:c.1672A>T		Nonsense		0.00009197	yes	48					
**31: M, 37**	Metabolic	*POLG*	ENST00000268124.11:c.2243G>C	Compound Het	Frameshift	Pathogenic	0.0007424	yes	32				3.37	no
			ENST00000268124.11:c.3528_3531del		Indel--> frameshift		0	yes						
**32: F, 74**	Spastic ataxia	*SPG7*	ENST00000645818.2:c.1529C>T	Hom	Splice acceptor	Pathogenic	0.003588	yes	25.4			1.2	3.45	no
**33: M, 82 (deceased)**	Sensory ataxia	*RFC1*	*RFC1*	Hom	Repeat expansion	Pathogenic		yes						no

**34: F, 51**	Metabolic	*POLR3A*	ENST00000372371.8:c.3718G>C	Compound Het	Splice region	Likely pathogenic	0.0001315	yes	7.99				-5.1	no
		POLR3A	ENST00000372371.8:c.1771-7C>G		Missense		0	no						
**35a: M, 32**	Metabolic	*GLS*	Exon 1 duplication	Hom	Structural variant-Dup	Pathogenic	0	yes						yes
**35b: M, 34**	Metabolic	*GLS*	Exon 1 duplication	Hom	Structural variant-Dup	Pathogenic	0	yes						
**36: F, 76**	Late complex	*ATXN3*	*ATXN3*	Het	Repeat expansion	Pathogenic		yes						no
**37: M, 73**	Metabolic	*NOP56*	*NOP56*	Het	Repeat expansion	Pathogenic		yes						no
**38: F, 78**	Sensory ataxia	*RFC1*	*RFC1*	Hom	Repeat expansion	Pathogenic		yes						no
**39: M, 30**	Early complex	*AIFM1*	ENST00000287295.8:c.784G>A	Hemi	Missense	Likely pathogenic	0	yes						no
**40: M, 75**	Sensory ataxia	*RFC1*	*RFC1*	Hom	Repeat expansion	Pathogenic		yes						no
**41: F, 73**	Sensory ataxia	*RFC1*	*RFC1*	Hom	Repeat expansion	Pathogenic		yes						no
**42: F, 38**	Early complex	*SETX*	ENST00000224140.6:c.4160_4161dup	Hom	Frameshift	Likely pathogenic	0	no						no
**43: F, 71**	Late complex	*RFC1*	*RFC1*	Hom	Repeat expansion	Pathogenic		yes						no
**44: M, 74**	Pure	*SPG7*	ENST00000645818.2:c.1529C>T	Hom	Splice acceptor	Pathogenic	0.003588	yes	25.4			1.2	3.45	no
**45: M, 34**	Metabolic	*AFG3L2*	ENST00000269143.8:c.2069G>T	Het	Missense	Likely pathogenic	0	no						no
**46: F, 53**	Sensory ataxia	*RFC1*	*RFC1*	Hom	Repeat expansion	Pathogenic		yes						no
**47: M 60**	Pure	*CACNA1A*	ENST00000360228.11:c.2636_2652dup	Het	Frameshift	Likely pathogenic	0	yes						no
**48: F, 51**	Sensory ataxia	*RFC1*	*RFC1*	Hom	Repeat expansion	Pathogenic		yes						no
**49: M, 75**	Episodic ataxia	*CACNA1A*	ENST00000360228.11:c.1165C>T	Het	Missense	Hot VUS[#]	0	yes	33	0	0.996		2.92	no
**50: F, 30**	Early complex	*SACS*	ENST00000382292.9:c.8612dup	Compound Het	Frameshift	Likely pathogenic	0.00006577	yes	34				4.75	no
		SACS	ENST00000382292.9:c.5891A>G		Missense	Likely pathogenic	0	no						
**51: F, 80**	Late complex	*CLN6*	ENST00000249806.11:c.712T>C	Compound Het	Missense	Pathogenic	0.00001971	yes	25.5			5.81	3.11	no
		CLN6	ENST00000249806.11:c.808C>T		Missense		0	no						
**52: F, 61**	Sensory ataxia	*FXN*	*FXN*	Hom	Repeat expansion	Pathogenic		yes						no
**53: M, 79 (deceased)**	Sensory ataxia	*RFC1*	*RFC1*	Hom	Repeat expansion	Pathogenic		yes						no
**54: M, 23**	Episodic ataxia	*CACNA1A*	ENST00000360228.11:c.5529-1270A>G	Het	Splice acceptor	Likely pathogenic	0	no						no

**55: F, 38**	Early complex	*SACS*	ENST00000382292.9:c.11707C>T	Compound Het	Nonsense	Pathogenic	0.00001315	yes	41			3.56	1.26	no
		SACS	ENST00000382292.9:c.13132C>T		Nonsense	Pathogenic	0	yes	42				0.31	
**56: M, 23**	Early complex	*CACNA1A*	ENST00000360228.11:c.4988G>A	Het	Missense	Pathogenic	0	yes	32				2.92	no
**57: F, 30**	Sensory ataxia	*FXN*	*FXN*	Hom	Repeat expansion	Pathogenic		yes						no
**58a: F, 56**	Spastic ataxia	*KCNC3*	ENST00000477616.2:c.1609G>A	Het	Missense	Hot VUS[#]	0	no	28.7					yes
**58b: M, 89**	Late complex	*KCNC3*	ENST00000477616.2:c.1609G>A	Het	Missense	Hot VUS[#]	0	no	28.7					
**59: F, 30**	Metabolic	*PNPLA6*	ENST00000600737.6:c.3061_3062insAGCC	Compound Het	Frameshift	Pathogenic	0.0001249	yes	33			6.35	2.1	no
			ENST00000600737.6:c.838C>G		Missense	Pathogenic	0.000006576	yes	24.1	0	0.761	6.78		
**60: M, 65**	Metabolic	*SPG7*	ENST00000645818.2:c.861dup	Compound Het	Structural variant-Dup	Likely pathogenic	0.00002628	yes	33					
		SPG7	ENST00000645818.2:c.1948G>A		Missense	Likely pathogenic	0.00005256	yes	27.4	0	0.998	1.71	3.36	no
**61: F, 68**	Spastic ataxia	*SPG7*	ENST00000645818.2:c.851del	Compound Het	Frameshift	Pathogenic	0	no						no
		SPG7	ENST00000645818.2:c.1529C>T		Splice acceptor	Pathogenic	0.003588	yes	25.4			1.2	3.45	
**62: M, 66**	Episodic ataxia	*CACNA1A*	ENST00000360228.11:c.4002del	Het	Frameshift	Likely pathogenic	0	no						no
**63: M, 25**	Early complex	*SHANK3*	ENST00000262795.8:c.1949del	Het	Frameshift	Likely pathogenic	0	no						no
**64: M, 51**	Late complex	*ATXN3*	*ATXN3*	Het	Repeat expansion	Pathogenic		yes						no
**65: M, 68**	Spastic ataxia	*SPG7*	ENST00000645818.2:c.1529C>T	Compound Het	Splice acceptor	Pathogenic	0.003588	yes	25.4			1.2	3.45	no
		SPG7	ENST00000645818.2:c.1053dup		Frameshift		0.0002366	yes	27.1			-0.8		
**66: M, 24**	Episodic ataxia	*ATXN6*	*ATXN6*	Het	Repeat expansion	Pathogenic		yes						no
**67: F, 26**	Episodic ataxia	*CACNA1A*	ENST00000360228.11:c.869A>G	Het	Missense	Likely pathogenic	0	no	27.5					no
**68: M, 36**	Early complex	*KCNC3*	ENST00000477616.2:c.1268G>A	Het	Missense	Pathogenic	0	yes	28.7	0	1		2.47	no
**69: M, 48**	Spastic ataxia	*SPG7*	ENST00000645818.2:c.1553-2_1553-1del	Hom	Splice acceptor	Pathogenic	0.00003284	yes	33				3.59	no
**70: M, 84**	Late complex	*GRM1*	ENST00000282753.6:c.785A>G	Het	Missense	Likely pathogenic	0	yes	28	0	0.999			yes
**71: M, 43**	Episodic ataxia	*CACNA1A*	ENST00000360228.11:c.2248C>A	Het	Missense	Likely pathogenic	0	no	33					no
**72a: M, 57**	Pure	*CACNA1A*	ENST00000360228.11:c.4034G>A	Het	Missense	Pathogenic	0	yes	31	0	0.945		2.13	yes
**72b: F, 20**	Pure	*CACNA1A*	ENST00000360228.11:c.4034G>A	Het	Missense	Pathogenic	0	yes	31	0	0.945		2.13	
**73a: M, 51**	Early complex	*KCND3*	ENST00000302127.5:c.1034G>T	Het	Missense	Likely pathogenic	0	yes	24	0	0.552		4	yes

**73b: M, 54**	Pure	*KCND3*	ENST00000302127.5:c.1034G>T	Het	Missense	Likely pathogenic	0	yes	24	0	0.552		4	
**74a: F, 63**	Pure	*KCND3*	ENST00000302127.5:c.1050G>T	Het	Missense	Hot VUS[#]	0	yes	28.8					yes
**74b: M, 39**	Late complex	*KCND3*	ENST00000302127.5:c.1050G>T	Het	Missense	Hot VUS[#]	0	yes	28.8					
**75: F, 43**	Metabolic	*OPA1*	ENST00000361510.8:c.*4_*5+2del	Hom	Splice donor	Likely pathogenic	0.00002628	yes	34					no
**76a: F, 6**	Early complex	*NKX2-1*	ENST00000354822.7:c.650C>A	Het	Nonsense	Pathogenic	0	yes	32	0	0.514		2.19	no
**76b: M, 45**	Early complex	*NKX2-1*	ENST00000354822.7:c.650C>A	Het	Nonsense	Pathogenic	0	yes	32	0	0.514		2.19	
**77: M, 34**	Sensory ataxia	*ATXN2*	*ATXN2*	Het	Repeat expansion	Pathogenic		yes						no
**78: F, 60 (deceased)**	Spastic ataxia	*ANO10*	ENST00000292246.8:c.132dup	Hom	Frameshift	Pathogenic	0.0006	yes	25.4			1.75		no
**79: M, 45**	Pure	*SYNE1*	ENST00000367255:c.26098C>T	Compound Het	Nonsense	Likely pathogenic	0.00000657	yes	59			2.44		no
		SYNE1	ENST00000367255:c.12528+1G>A		Splice donor variant	Likely pathogenic	0.0000131	yes	31			2.29		
**80: F, 58**	Episodic ataxia	*CACNA1A*	ENST00000360228.11:c.4996C>T	Het	Missense	Pathogenic	0	yes	29.2	0				no
**81: F, 76**	Spastic ataxia	*ANO10*	ENST00000292246.8:c.132dup	Hom	Frameshift	Likely pathogenic	0.0006	yes	25.4			1.75		no
**82: M, 33**	Spastic ataxia	*SPG7*	ENST00000645818.2:c.2084T>C	Compound Het	Frameshift	Pathogenic	0.00001314	yes	29.7			1.71		no
		SPG7	ENST00000645818.2:c.1454_1462del		Missense	Pathogenic	0.0004022	yes					3.45	
**83: M, 66**	Spastic ataxia	*TTBK2*	ENST00000267890.11:c.1297_1304del	Het	Frameshift	Likely pathogenic	0	no						no
**84: F, 46**	Spastic ataxia	*GLRA1*	ENST00000274576.9:c.737G>A	Het	Missense	Likely pathogenic	0.000006575	yes	26.9	0.001			3.65	no
**85: F, 71**	Sensory ataxia	*RFC1*	*RFC1*	Hom	Repeat expansion	Pathogenic		yes						no
**86: M, 65**	Spastic ataxia	*MT_ATP6*	ENST00000361899:c.650T>C	mito heteroplasmy 99.6%	Missense	Pathogenic		yes						no
**87: M 64**	Late complex	*CACNA1A*	ENST00000360228.11:c.1594G>A	Het	Missense	Pathogenic	0.000006575	yes	29.5	0			1.03	no
**88: F, 53**	Metabolic	*CAPN1*	ENST00000279247.11:c.337+1G>A	Hom	Splice donor	Pathogenic	0	no	33	0	1		3.2	no
**89: M, 54**	Metabolic	*POLR3A*	ENST00000372371.8:c.3797C>T	Compound Het	Missense	Likely pathogenic	0	no	27	0.04	0.702			no
		POLR3A	ENST00000372371.8:c.685C>T		Nonsense	Pathogenic	0.0000131	yes	36					
**90: F, 40**	Late complex	*SPG21*	ENST00000204566.7 c.152_153del	Hom	Nonsense	Likely pathogenic	0	yes						no
**91: M, 56**	Late complex	*SPG7*	ENST00000645818.2:c.1529C>T	Compound Het	Missense	Likely pathogenic	0.003588	yes	25.4			1.2	3.45	no
		SPG7	ENST00000645818.2:c.1904C>T		Missense	Pathogenic	0	yes						

**92: F, 55**	Late complex	*PRKCG*	ENST00000263431(PRKCG-201):c.413T>A	Het	Missense	Likely pathogenic	0.000006577	yes	26.5	0			4.44	no
**93: M, 46**	Late complex	*SYNE1*	ENST00000367255.10:c.14500C>T	Compound Het	Nonsense	Pathogenic	0.00000657	yes	37				2.98	no
		SYNE1	ENST00000367255.10:c.14290C>T		Nonsense		0.00001972	yes	37				2.98	
**94: M, 72**	Late complex	*ANO10*	ENST00000292246.8:c.59del	Compound Het	Frameshift	Pathogenic	0	no	26.7				2.19	yes
		ANO10	ENST00000292246.8:c.1258_1259delinsTT		Missense	Hot VUS[#]	0	no	27.1					
**95: F, 90 (deceased)**	Sensory ataxia	*RFC1*	*RFC1*	Hom	Repeat expansion	Pathogenic		yes						no
**96: M, 64**	Late complex	*SPG7*	ENST00000645818.2:c.1529C>T	Hom	Splice acceptor	Pathogenic	0.003588	yes	25.4			1.2	3.45	no
**97: F, 76**	Spastic ataxia	*MT_ATP6*	ENST00000361899.2:c.257G>A	mito heteroplasmy 99.8%	Missense	Pathogenic		yes						no
**98: M, 54**	Metabolic	*SPG7*	ENST00000645818.2:c.233T>A	Hom	Nonsense	Pathogenic	0.0001997	yes	35				-3.31	no
**99a: M, 43**	Spastic ataxia	*ACBD5*	ENST00000396271.8:c.729_732del	Hom	Nonsense	Likely pathogenic	0	no						yes
**99b: M, 50**	Metabolic	*ACBD5*	ENST00000396271.8:c.729_732del	Hom	Nonsense	Likely pathogenic	0	no						
**100: F, 40**	Metabolic	*SPG7*	ENST00000645818.2:c.1529C>T	Hom	Splice acceptor	Pathogenic	0.003588	yes	25.4			1.2	3.45	no
**101: F, 59**	Metabolic	*PRKCG*	ENST00000263431.4:c.197G>A	Het	Missense	Pathogenic	0	yes	27.3	0	0.98		2.01	no
**102: M, 73**	Pure	*RFC1*	*RFC1*	Hom	Repeat expansion	Pathogenic		yes						no
**103: F, 80**	Sensory ataxia	*RFC1*	*RFC1*	Hom	Repeat expansion	Pathogenic		yes						no
**104: M, 43**	Spastic ataxia	*SPG7*	ENST00000645818.2:c.1046_1047insC	Hom	Frameshift	Likely pathogenic	0	yes					3.75	no
**105: M, 72**	Late complex	*CACNA1G*	ENST00000359106.10:c.3272G>T	Het	Missense	Likely pathogenic	0	no	31	0	0.931		2.09	no
**106: M, 75**	Pure	*KCNC3*	ENST00000477616.2:c.1746_1754del	Het	Disruptive inframe deletion	Hot VUS[#]	0.00009856	yes	15.9				2	no
**107: F, 50**	Pure	*SPTBN2*	ENST00000533211.6:c.7004_7012del	Het	Disruptive inframe deletion	Likely pathogenic	6.574E-07	no					2.42	no
**108: M, 70**	Late complex	*ITPR1*	ENST00000649015.2:c.4346A>C	Het	Missense	Likely pathogenic	0	no	31					yes
**109: M, 31**	Pure	*CACNA1G*	ENST00000359106.10:c.1177G>A	Het	Missense	Hot VUS[#]	0	no	29					yes
**110: F, 56**	Pure	*PRKCG*	ENST00000263431.4:c.172G>T	Het	Missense	Likely pathogenic	0	no	26.7				2.01	yes
**111: M, 69**	Spastic ataxia	*SPG7*	ENST00000645818.2:c.1048C>A	Compound Het	Nonsense	hot VUS[#]	0.0001511	no	23.2	0	0.999	-0.8		no
		SPG7	ENST00000645818.2:c.1529C>T		Splice acceptor	Likely pathogenic	0.003588	yes	25.4			1.2	3.45	

**112: F, 65**	Metabolic	*RP1L1*	ENST00000382483.4:c.4294_4295insGGCCAGGAGGAGGAAG	Het	Frameshift	Likely pathogenic	0	no						no
**113: M, 59**	Metabolic	*SPG7*	ENST00000645818.2:c.1529C>T	Compound Het	Splice acceptor	Likely pathogenic	0.003588	yes	25.4			1.2	3.45	yes
		SPG7	ENST00000645818.2:c.1715C>T		Missense	Likely pathogenic	0.0001511	yes	24.4				3.53	
**114: F, 68**	Sensory ataxia	*RFC1*	*RFC1*	Hom	Repeat expansion	Pathogenic		yes						no
**115: F, 52**	Spastic ataxia	*SPG7*	*SPG7* complex deletion exons 13-17/17	Hom	Structural variant-Del	Likely pathogenic	0	no						no
**116: F, 78**	Sensory ataxia	*RFC1*	*RFC1*	Hom	Repeat expansion	Pathogenic		yes						no
**117: M, 62**	Metabolic	*ECHS1*	ENST00000368547.4:c.518C>T	Compound Het	Missense	Likely pathogenic	0.0001248	yes	22.5	0.034				yes
			ENST00000368547.4:c.299T>C		Missense	Likely pathogenic	0	yes	29.1	0				
**118: F, 62**	Metabolic	*ANO10*	*ANO10* deletion of exon 12/13	Compound Het	Structural variant-Del	Likely pathogenic	0	no						no
**119: F, 63**	Metabolic	*ERCC6*	ENST00000355832.10:c.1820A>T	Compound Het	Missense	Pathogenic	0	yes	34	0	0.987	0.64	3.78	yes
		ERCC6	ENST00000355832.10:c.2167C>T		Nonsense	Pathogenic	0.00008557	yes	50				2.47	
**120: F, 73**	Metabolic	*GBE1*	ENST00000429644.7:c.986A>C	Hom	Missense	Hot VUS#	0.0001908	yes	28.9			-0.3		no
**121: M, 69**	Spastic ataxia	*POLR3A*	ENST00000372371.8:c.685C>T	Compound Het	Nonsense	Pathogenic	0	no	36				0.33	no
		POLR3A	ENST00000372371.8:c.1909+22G>A		Splice region	Pathogenic	0.001544	yes						
**122: M, 72**	Episodic ataxia	*PRKCG*	ENST00000263431.4:c.1844G>C	Het	Missense	Hot VUS#	0	no	26.1				2.13	no

Genetic variants of seven proband remained as variants of unknown significance but with high suspicion of pathogenicity, otherwise known as 'hot VUS' based on ACMG criteria.(Houge *et al.*, 2022) These were *CACNA1A, KCNC3, KCND3, CACNA1G, GBE1 and PRKCG* (etable 2). No additional family members were available for segregation. We were not able to acquire additional tissue samples for functional validation.

Reference:

Houge G, Laner A, Cirak S, de Leeuw N, Scheffer H, den Dunnen JT. Stepwise ABC system for classification of any type of genetic variant. Eur J Hum Genet 2022; 30(2): 150-9.

Supplementary Table 3. Ataxia clinical subgroups and type of genetic variants in probands with confirmed molecular diagnoses

Genetic Variants	Pure (N=10)	Spastic (N=25)	Sensory (N=20)	Metabolic (N=24)	Early complex (N=13)	Late complex (N=16)	Episodic (N=7)	Total and percentage of probands solved (N=115)
**Single nucleotide variant**	6 (60%)	13 (52%)	0 (0%)	19 (79%)	10 (77%)	8 (50%)	4 (57%)	60 (52%)
**Mitochondrial variant**	0 (0%)	2 (8%)	0 (0%)	0 (0%)	0 (0%)	0 (0%)	0 (0%)	2 (2%)
**Indel**	2 (20%)	7 (28%)	0 (0%)	2 (8%)	2 (15%)	2 (13%)	1 (14%)	16 (14%)
**SV deletion**	0 (0%)	1 (4%)	0 (0%)	1 (4%)	0 (0%)	0 (0%)	0 (0%)	2 (2%)
**SV duplication**	0 (0%)	1 (4%)	0 (0%)	1 (4%)	0 (0%)	0 (0%)	1 (14%)	3 (3%)
**Repeat expansion**	2 (20%)	1 (4%)	20 (100%)	1 (4%)	1 (8%)	6 (38%)	1 (14%)	32 (28%)

Supplementary Table 4. Analysis of relationship between age of disease onset, ataxia clinical subtype, family history and whether the proband achieved a genetic diagnosis using logistic regression

A. Univariate Analysis of the factors that influence the diagnostic rate in all probands

Age at Onset (AAO)	Solved (N=114)*	Unsolved (N=234)*
<30 years$	44 (39%)	68 (29%)
30 years and above	70 (61%)	166 (71%)

Conclusion:
The odds of patients aao>30 achieving a diagnosis is 0.65 times that of the odds of patients with aao<30 (CI: 0.41-1.05, p=0.07)

Family history	Solved (N=115)	Unsolved (N=235)$
Familial%	56 (49%)	70 (30%)
Non-familial	59 (51%)	165 (70%)

Conclusion:
The odds of patients without family history achieving diagnosis is 0.45 than that of the odds of patient with family history achieving the diagnosis (CI: 0.28-0.71, P=0.0005)

*Three probands had missing AAO data
$One proband had missing family history data

B. Multivariate Analysis of the factors that influence diagnostic rate in all probands

Coefficients:	Estimate	Std. Error	z value	Pr(>|z|)
Intercept	-0.196575	0.519019	-0.379	0.7049
Aao ≥30	0.245137	0.329773	0.743	0.4573
Early complex	-0.052540	0.627622	-0.084	0.9333
Late complex	-0.006611	0.588170	-0.011	0.9910
metabolic	0.485921	0.563155	0.863	0.3882
Pure	-1.461920	0.592203	-2.469	0.0136 *
Sensory ataxia	1.492889	0.629886	2.370	0.0178 *
Spastic ataxia	0.540565	0.556980	0.971	0.3318
No family history	-1.028444	0.262554	-3.917	8.96e-05 ***

Bonferroni correct P<0.02
Hosmer and Lemeshow goodness of fit (GOF) test: X-squared = 3.2146, df = 8, p-value = 0.9202

Conclusions:
The odds of achieving a genetic diagnosis in individuals with pure ataxia using WGS is only 9% compared to individuals with other ataxia subtypes (95% CI: 2.7%-27%, p=0.0136).
The odds of achieving a genetic diagnosis in individuals without a family history is only 43% compared to individuals with a family history (95% CI: 26%-70%, p<0.0001)

The odds of achieving a genetic diagnosis in individuals with sensory ataxia is 70% more likely than individuals with other ataxia subtypes (95% CI: 52%-84%, $p=0.0178$)

Supplementary Table 5. Putative genes diagnosed via WGS and their frequency in ataxia clinical subgroups (n=115)

Genes	Pure Ataxia	Spastic Ataxia	Sensory Ataxia	Ataxia with Metabolic Features	Early Complex Ataxia	Late Complex Ataxia	Episodic Ataxia	Total per gene
ACBD5	0	1	0	0	0	0	0	1
ACP33	0	0	0	0	0	1	0	1
AFG3L2	0	0	0	1	0	0	0	1
AIFM1	0	0	0	0	1	0	0	1
ANO10	0	2	0	1	0	1	0	4
AR	0	0	0	0	1	0	0	1
ATN1	0	0	0	0	0	1	0	1
ATXN2	0	0	1	0	0	0	0	1
ATXN3	0	0	0	0	0	2	0	2
ATXN6	1	0	0	0	0	0	1	2
CACNA1A	2	0	0	0	1	1	6	10
CACNA1G	0	0	0	0	0	1	0	1
CAPN1	0	0	0	1	0	0	0	1
CLN6	0	0	0	0	0	1	0	1
ECHS1	0	0	0	1	0	0	0	1
ELOVL4	1	0	0	0	0	0	0	1
ERCC6	0	0	0	1	0	0	0	1
FGF14	0	0	0	0	0	0	0	0
FXN	0	1	2	0	0	0	0	3
GBA2	0	0	0	0	1	0	0	1
GLRA1	0	1	0	0	0	0	0	1
GLS	0	0	0	1	0	0	0	1
GRM1	0	0	0	0	0	1	0	1
ITPR1	0	0	0	0	0	1	0	1

KCNC3	1	0	0	0	1	0	0	2
KCND3	0	0	0	0	1	0	0	1
MT_ATP6	0	2	0	0	0	0	0	2
NKX2_1	0	0	0	0	1	0	0	1
NKX6_2	0	0	0	0	1	0	0	1
NOP56	0	0	0	1	0	0	0	1
NPC1	0	0	0	0	1	0	0	1
OPA1	0	0	0	2	0	0	0	2
PNPLA6	0	0	0	1	0	0	0	1
POLG	0	0	0	1	0	0	0	1
POLR3A	0	1	0	2	0	0	0	3
PPP2R2B	0	0	0	0	0	1	0	1
PRKCG	1	0	0	1	0	1	0	3
RFC1	1	0	17	0	0	2	0	20
RNF216	0	0	0	1	0	0	0	1
RP1L1	0	0	0	1	0	0	0	1
SACS	0	0	0	2	1	0	0	3
SETX	0	1	0	0	1	0	0	2
SHANK3	0	0	0	0	1	0	0	1
SPG7	1	13	0	6	0	2	0	22
SPTBN2	1	0	0	0	0	0	0	1
SYNE1	1	2	0	0	1	0	0	4
TTBK2	0	1	0	0	0	0	0	1
**Total per clinical subgroup**	13	25	20	24	13	18	12	115

Supplementary Table 6. Examples illustrating importance of phenotyping and HPO term inclusion in selecting correct gene panels on Genomic England PanelApps

**Proband ID**	Gene	Ataxia subgroup	Key additional HPO terms	Gene panels added that led to molecular Dx
39	*AIFM1*	Early complex	HP:0000407 sensorineural hearing impairment<br>HP:0002936 distal sensory impairment<br>HP:0011463 childhood onset<br>HP:0007141 sensorimotor neuropathy<br>HP:0001284 areflexia<br>HP:0002751 kyphoscoliosis<br>HP:0002194 delayed gross motor development	Hereditary neuropathy
63	*SHANK3*	Early complex	HP:0000750 delayed speech and language development<br>HP:0003621 juvenile onset<br>HP:0001249 intellectual disability	Intellectual disability
84	*GLRA1*	Spastic	HP:0001336 myoclonus<br>HP:0002267 exaggerated startle response<br>HP:0001257 spasticity	Brain channelopathy
99	*ACBD5*	Spastic	HP:0000007 autosomal recessive inheritance<br>HP:0000648 optic atrophy<br>HP:0003429 CNS hypomyelination	White matter disorders and cerebral calcification-narrow panel; inherited white matter
112	*RP1L1*	Metabolic	HP:0000505 visual impairment<br>HP:0007703 abnormality of retinal pigmentation	Retinal disorders
117	*ECHS1*	Metabolic	HP:0000007 autosomal recessive inheritance<br>HP:0000365 hearing impairment<br>HP:0000648 optic atrophy<br>HP:0002194 delayed gross motor development	Undiagnosed metabolic disorders
119	*ERCC6*	Metabolic	HP:0002072 chorea<br>HP:0001257 spasticity<br>HP:0430048 intracranial calcification<br>HP:0000988 skin rash<br>HP:0000992 cutaneous photosensitivity<br>HP:0025331 upgaze palsy<br>HP:0003487 babinski sign	Xeroderma pigmentosum, Trichothiodystrophy or Cockayne syndrome; White matter disorders and cerebral calcification-narrow panel

Supplementary Table 7. Results of GAA-FGF14 testing using long-range PCR, repeat-primed PCR and Sanger sequencing in probands with GAA repeat expansion above pathogenic threshold (250 repeats).

ID; sex; age	Ataxia subgroup	Repeat Size 1	Repeat Size 2
**123: F, 74**	Pure	110	280
**124: M, 75**	Pure	206	301
**125: M, 77**	Late complex	8	426
**126: F, 54**	Pure	9	427
**127: M, 81**	Late complex	113	380
**128: M, 68**	Episodic ataxia	16	351
**129: M, 84**	Episodic ataxia	93	319
**130: M, 64**	Episodic ataxia	16	269
**131: F, 77**	Episodic ataxia	107	370
**132: M, 76**	Episodic ataxia	15	395
